# GCN2 phosphorylates HIV-1 integrase and decreases HIV-1 replication by limiting viral integration

**DOI:** 10.1038/s41598-017-02276-0

**Published:** 2017-05-23

**Authors:** A. Jaspart, C. Calmels, O. Cosnefroy, P. Bellecave, P. Pinson, S. Claverol, V. Guyonnet-Dupérat, B. Dartigues, M. S. Benleulmi, E. Mauro, P. A. Gretteau, V. Parissi, M. Métifiot, M. L. Andreola

**Affiliations:** 1 Université de Bordeaux, CNRS UMR 5234, MFP, 146 rue Léo Saignat, 33076 Bordeaux cedex, France; 2 Fédération de Recherche “TransbioMed”, Bordeaux, France; 3 Laboratoire de Virologie. CHU de Bordeaux, Bordeaux, France; 4 Université de Bordeaux, Centre Génomique Fonctionnelle de Bordeaux, Plateforme Protéome, Bordeaux F-33000 France; 5 INSERM 217 Plateforme vectorologie, Bordeaux, France; 6 Centre de Bioinformatique de Bordeaux – CGFB- Université Bordeaux Segalen, 146 rue Léo Saignat, 33076 Bordeaux Cedex, France; 7 Laboratoire de Virologie Médicale et Moléculaire (EA CardioVir), Reims, France

## Abstract

GCN2 is a serine/threonine kinase involved in cellular stress response related to amino acid starvation. Previously, we showed that GCN2 interacts with HIV-1 integrase and is activated during HIV-1 infection. Herein, we identified HIV-1 integrase as a previously unknown substrate of GCN2 *in vitro* with a major site of phosphorylation at residue S255 located in the C-terminal domain of HIV-1 integrase. The underlying mechanism was investigated and it appeared that the integrase active site was required in order for GCN2 to target the integrase residue S255. Moreover, various integrases from other retroviruses (*e*.*g*. MLV, ASV) were also recognized as a substrate by GCN2. In cells, HIV-1 lentiviral particles harboring mutation at integrase position 255 were affected in their replication. Preventing phosphorylation resulted in an increase in infectivity that correlated with an increase in viral DNA integration. Infectivity of MLV was also higher in cells knocked-out for GCN2 suggesting a conserved mechanism to control viral replication. Altogether, our data suggest that GCN2 may constitute a general guardian of genome stability by regulating foreign DNA integration and as such be part of the antiviral armamentarium of the cell.

## Introduction

**Figure 1:**
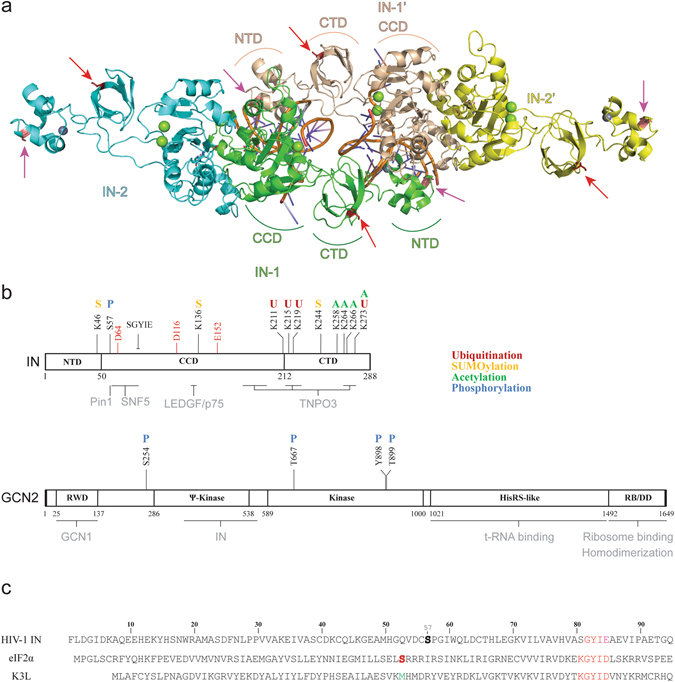
Structure of HIV-1 IN and GCN2. (**a**) Model of the HIV-1 intasome structure. A tetramer of IN is represented as cartoon colored by subunits, with 2 viral DNA ends colored by element. The magnesium and zinc ions are represented as green and grey spheres, respectively. The full-length structure of monomers derives from the model published by Johnson *et al*.^14^. The catalytic and structural monomers are indicated as IN-1/IN-1′ and IN-2/IN-2′, respectively. (**b**) Schematic organization of HIV IN (top) and human GCN2 (bottom). Regions known to participate in cofactor binding are highlighted in grey below the scheme. Post-translational modifications that have been described in the literature are reported above the scheme and color coded. The IN catalytic triad DDE is highlighted in red. (**c**) Alignment of the N-terminal sequence of HIV-1 IN, human eIF2α and of the vaccinia virus protein K3L.

Retroviruses are RNA viruses relying for their replication on the reverse transcription of their genome to double stranded DNA and on the production of provirus copies integrated into the host cell genome. This integration step is catalyzed by a virally encoded integrase (IN). Accumulated biochemical and structural data give an overview of the enzymatic reaction taking place^1^. IN associates itself with the viral DNA to form the intasome, composed of at least a tetramer of enzyme with two viral DNA ends. From this oligomer (Fig. 1a), only two monomers bind the extremity of the viral DNA and sustain activity. The other subunits present a structural role and are thought to hold the complex in proper conformation. HIV-1 IN is a 32 kDa protein sharing common properties with other retroviral INs^2^. It has an N-terminal domain (1–50, NTD) with a H_12_H_16_C_40_C_43_ motif and a C-terminal domain containing a SH3 fold (212–288, CTD). The active site is encompassed within the central domain (catalytic core domain, CCD), and is composed of a catalytic triad D_64_D_116_E_152_ that coordinates two magnesium ions (Fig. 1b).

Regarding catalysis, IN first hydrolyzes the viral DNA from the 3′-ends (3′-processing, 3′-P) and uses the newly created 3′-OH extremities to attack the phosphodiester backbone of the target DNA (strand transfer, ST)^3^. While IN alone is sufficient to integrate viral DNA mimics *in vitro*, the cellular process involves the formation of a pre-integration complex or PIC composed of viral and cellular proteins. Various studies focused on identifying proteins able to interact with IN^3^. These cofactors have multiple roles, including stabilization of the complex, tethering to the integration site and regulation of IN activity^4^. This regulation also involves post-translational modifications of the intasome and IN has been shown to undergo acetylation, phosphorylation, SUMOylation and ubiquitination (Fig. 1b)^5^. Interestingly, IN phosphorylation at position S_57_ by the cellular c-Jun N-terminal kinase (JNK) enables its binding to Pin1 (a peptidyl prolyl-isomerase) and seems to interfere with ubiquitination^6^. Interaction with Pin1 increases IN stability and ultimately is required for efficient viral replication in activated T cells.

Using yeast two-hybrid and co-immunoprecipitation experiments, we identified GCN2 (general control nonderepressible 2), another serine/threonine protein kinase as a novel IN interacting cellular factor. Interestingly, we found GCN2 to be activated upon HIV-1 infection which, in turn, decreased HIV-1 replication^7, 8^. This protein kinase is conserved from yeast to human and is part of the four eIF2α’s kinases found in mammalian cells along with PERK, PKR and HRI. Human GCN2 is a 187 kDa protein sensor involved in cellular stress response related to amino acid starvation (Fig. 1b)^9, 10^. It harbors a RWD domain in N-terminal responsible for GCN1 binding^11^. The 286–538 region is a pseudo-kinase domain (YKD) which interaction with the kinase domain (KD, 589–1000) is important in the activation process. The C-terminal portion is homologous to histidyl-tRNA synthetase (HisRS), contains a ribosome binding motif and is also involved in homodimerization^12^. When the level in amino acid reaches a critical limit in the cell, uncharged tRNAs bind to the HisRS-like domain and induce a conformational change resulting in GCN2 autophosphorylation at conserved threonine residues (pGCN2)^10^. Similarly, interaction of the GCN1/GCN20 complex to the N-terminal domain of GCN2 facilitates binding of uncharged tRNA and subsequent activation.

Once activated, GCN2 phosphorylates the subunit one of the heterotrimer eukaryotic initiation factor 2 (eIF2α) leading to stabilization of the inactive GDP complex. As a result, eIF2-GDP is unable to load methionyl-tRNA onto the ribosome^13^. On a biochemical point of view, GCN2 recognizes a conserved _79_KGYID_83_ motif on eIF2α and transfers the γ-phosphoryl group of an ATP molecule to residue S_51_ of eIF2α. A strong sequence homology has been found with the vaccinia virus protein K3L^14^ (Fig. 1c). However, K3L lacks a phosphorylatable residue at position 51 and thus acts as a pseudo-substrate, inhibiting phosphorylation of eIF2α.

Interestingly, our previous study showed that direct delivery of recombinant HIV-1 IN protein into amino acids starved cells prevented GCN2 phosphorylation and tended to reduce the protein synthesis arrest^8^. Because IN binds GCN2 through its pseudo-kinase domain^8, 15^, IN might interfere with the YKD/KD interactions required during the kinase activation process. Thus, interferences observed in GCN2 signaling could be the result of IN being recognized as a pseudo-substrate, similarly to K3L^14^.

In the present work, we studied *in vitro* the mechanism by which HIV-1 IN could interfere with GCN2 kinase activity and showed that HIV-1 IN, as well as IN from other retroviruses were indeed substrates for GCN2. Point mutations were designed to impede phosphorylation and were introduced in the IN coding sequence of lentiviral particles. Infectivity of such mutant viruses was compared to wild-type (WT) HIV-1 and the viral step affected by the mutation was determined.

## Materials and Methods

### Recombinant Proteins

GCN2 was purchased from SignalChem (cat# E12-11G). This recombinant protein was expressed in insect cells as a truncated hyper-activated mutant (192–1024) with an N-terminal GST tag (132 kDa). HIV-1 IN WT and phosphorylation mutants (S24A, S24D, S255A, S255D, S24A/S255A, and S24D/S255D) were expressed and purified as previously described^16^. IN expressed in yeast was purified as described by Faure *et al*., in ref. 17. Truncated forms of HIV-1 IN (ΔNTD, 50–288; ΔCTD, 1–212; CCD, 50–212) were purified as previously described^18^. The HIV-1 IN isolated CTD was expressed in bacteria as a fusion protein with a N-terminal GST. After cell lysis [50 mM HEPES pH 7.5, 500 mM NaCl, 1 mM dithiothreitol (DTT), 1 mM phenylmethylsulfonyl fluoride (PMSF)], the soluble fraction was applied to a His SpinTrap column and retained proteins were further purified on a GST SpinTrap™ column (GE Healthcare). Elution was performed using steps at 500 mM imidazole and 20 mM reduced glutathione, respectively. HIV-1 IN harboring mutation Q168L and Q188Q were generous gifts from Dr. S. Emiliani (Cochin Institute, Paris, France). Vectors for the bacterial expression of eIF2α and exotic INs (Ty3 and chimers) were kind gifts from Dr. Sandmeyer. The Ty3 retrotransposon IN and the chimeric protein HPH (containing a CCD corresponding to PFV IN surrounded by the NTD and CTD of HIV-1 IN) were purified according to previous publication^19^. Cellular eIF2α was expressed and purified as previously reported^20^. Briefly, eIF2α was expressed in BL21 cells from a pET15b vector with a N-terminal 6xHis tag and a C-terminal deletion (lacking amino acids 200–304). The resulting ≈28 kDa protein was purified through a nickel affinity column using an AKTÄ purifier (GE Healthcare). Proteins purity was evaluated by Coomassie staining of a polyacrylamide gel electrophoresis and concentration was determined using a NanoDrop 2000 (ThermoScientific).

### *In vitro* phosphorylation assay

Phosphorylation of target proteins was achieved by incubating the substrate (700 nM, unless indicated otherwise) with GCN2 at 24 nM in a reaction buffer containing a mix of 10 mM Tris [2-amino-2-hydroxymethyl-1,3-propanediol]/20 mM HEPES [4-(2-hydroxyethyl)-1-piperazineethanesulfonic acid] buffered at pH 7.5, 50 mM NaCl, 15 mM MgCl_2_, 7.5 mM MnCl_2_, 10 mM dithiothreitol (DTT), 0.05% Nonidet P-40, 100 µM ATP, 50 µCi/µl of [γ-^32^P]-ATP (3000 Ci/mmole, Perkin Elmer). Phosphorylation was performed at 30 °C for 60 minutes unless indicated otherwise. Reaction (20 µl) was stopped by adding 10 µl of loading buffer [80 mM Tris-HCl pH 6.8, 100 mM DTT, 10% glycerol, 1% sodium dodecyl sulfate (SDS) and 0.05% bromophenol blue]. Samples were then loaded onto a denaturating SDS-PAGE. After protein separation (120 V for 90 minutes) the gel was stained using Coomassie Brilliant Blue R-250. Autoradiography was performed using an imaging plate (Fujifilm) and images were obtained with a FLA-5000 Imaging System (Fujifilm).

### Integrase catalytic activity

Kinetic of 3′-P and ST activities were performed as previously described^21^. Briefly, WT IN or mutant proteins (400 nM) were mixed with 20 nM radiolabeled DNA substrates in a buffer containing 50 mM MOPS pH 7.2, 7.5 mM MgCl_2_, 14 mM 2-mercaptoethanol at 37 °C. Reactions were stopped after 10, 20, 30, 45, 60, 90 or 120 minutes by addition of an equal volume of loading buffer (formamide containing 1% SDS, 0.25% bromophenol blue, and xylene cyanol). Reaction products were separated in 16% poly-acrylamide denaturing sequencing gels. Image of the gels was obtained after autoradiography using a FLA-5000 (Fujifilm). Densitometric analyses were performed using ImageQuant 5.1 software from GE Healthcare.

### Mass spectrometry

#### Sample preparation and protein digestion

Bands were cut from the SDS-PAGE gel and were unstained in 25 mM ammonium bicarbonate 50% ACN, rinsed twice in ultrapure water and shrunk in acetonitrile (ACN) for 10 min. After ACN removal, gel pieces were dried at room temperature, covered with trypsin solution (10 ng/µl in 40 mM NH_4_HCO_3_ and 10% ACN), rehydrated at 4 °C for 10 min, and finally incubated overnight at 37 °C. Gel pieces were then incubated for 15 min in 40 mM NH_4_HCO_3_ and 10% ACN at room temperature. The supernatant was collected, and the gel slices were bathed in an H_2_O/ACN/HCOOH (47.5:47.5:5) extraction solution for 15 min. The extraction step was repeated twice. The supernatants were dried in a vacuum centrifuge and resuspended in 100 µL of 0.1% HCOOH.

#### nLC-MS/MS analysis

The peptide mixture was analyzed on a Ultimate 3000 nanoLC system (Dionex, Amsterdam, The Netherlands) coupled to a LTQ Orbitrap XL mass spectrometer (ThermoFinnigan, San Jose, CA). Ten microliters of peptide digests were loaded onto a 300-µm-inner diameter × 5-mm C_18_ PepMap^TM^ trap column (LC Packings) at a flow rate of 20 µl/min in buffer A (0.1% formic acid, 5% ACN). Peptides were eluted from the trap column onto an analytical 75-mm id × 15-cm C18 Pep-Map column (LC Packings) with a 2–40% linear gradient of solvent B (0.1% formic acid, 80% ACN) in 108 min (200 nl/min). The mass spectrometer operated in positive ion mode at a 1.8-kV needle voltage. Data were acquired using the Xcalibur 2.0.7 software in a data-dependent mode. MS scans (*m*/*z* 300-2000) were recorded in the Orbitrap cell at a resolution of R = 60 000 (@ m/z 400) and an AGC target of 5 × 10^5^ ions collected within 500 ms. Dynamic exclusion was set to 30 s and the top 6 ions were selected from fragmentation in Collision Induced Dissociation (CID) mode. MS/MS scans with a target value of 1 × 10^4^ ions were collected in the linear ion trap with a maximum fill time of 200 ms. Additionally, unassigned and +1 charge state ions were rejected for fragmentation. Others settings were as follows: spray voltage, 1.8 kV, no sheath nor auxiliary gas flow, heated capillary temperature, 200 °C; normalized collision energy of 35% and an isolation width of 2 *m*/*z*.

#### Database search and results processing

Spectra originating from peptides with molecular weight below 350 and above 5000 Da were discarded. Selected peptides were then searched by SEQUEST through Proteome Discoverer 1.4 (Thermo Fisher Scientific Inc.) against the Human Reference Proteome Set (UniProt) database in which homemade sequences corresponding to IN were added. Mass accuracy of the monoisotopic peptide precursor and of the peptide fragments were set to 10 ppm and 0.6 Da, respectively. Only b- and y-ions were considered for mass calculation. The oxidation of methionine residues (+16 Da) and phosphorylation of serine, threonine and tyrosine residues (+80 Da) were considered as variable modification while carbamidomethylation of cysteine residues (+57 Da) as fixed modification. Two missed trypsin cleavages were allowed. Peptide validation was performed using the Percolator algorithm^22^, and only peptides with a 1% false positive rate at peptide level were considered of “high confidence” and subsequently retained. Phosphosite precise localization was determined using PhosphoRS 3.1 node embedded in Proteome Discoverer^23^.

### Site-directed mutagenesis

Site-directed mutagenesis was performed on the pET-21b-IN, pCMVΔR8.91, and pEGFP-C2-INs plasmids following a standard PCR-based protocol using degenerated primers obtained from MWG Biotech. The amplification reaction was performed using a step at 98 °C for 30 seconds, followed by 20 cycles of 10 seconds at 98 °C, followed by 45 seconds at 60 °C and 1 minute per kb at 72 °C; and finally a step of 10 minutes at 72 °C. Amplification products were subjected to *Dpn*I digestion for 1 hour at 37 °C (1 µl/50 µl reaction). DH5α chemically competent cells were transformed with the digestion product. After plasmid extraction (standard miniprep from Macherey-Nagel following manufacturer’s instructions), the presence of the desired mutations and the integrity of the IN sequence were verified by DNA sequencing (MWG Eurofins).

### Expression of IN in human cells

The plasmid for the expression of HIV-1 IN fused to the EGFP (pEGFP-C2-INs) was a gift from Dr. Z. Debyzer (KU Leuven)^24^. 293 T cells were plated in a 6-well culture plate (50,000 cells/well) and maintained in DMEM Glutamax® medium (Invitrogen, Carlsbad, CA) supplemented with 10% (v/v) heat-inactivated fetal calf serum (FCS, Invitrogen) and 50 µg/ml gentamicin (complete DMEM) at 37 °C with 5% CO_2_. After 24 h, cells were washed and transfected with 1 µg of pEGFP-C2-INs plasmid and 1 µl Lipofectamine 2000 reagent (Invitrogen) in 100 µl of optiMEM for 4 h. OptiMEM containing 20% FCS was added (100 µl/well) and cells were incubated for 20 h. After trypsinization, cells were lysed by incubation for 20 min at 4 °C in lysis buffer [20 mM Tris HCl pH 7.5; 50 mM NaCl; 2 mM EDTA; 1% Triton X-100, protease and phosphatase inhibitors (Roche)].

For immunoprecipitation, cell lysates were sonicated twice at 40 W for 20 seconds before the addition of the anti-EGFP antibody (mouse monoclonal, Clontech, dilution 1/500) in the presence of 50 µl of magnetic beads conjugated to sheep anti-mouse antibody (Dynabeads, Invitrogen) and incubated overnight at 4 °C. After 3 washes with phosphate-buffered saline (PBS; 140 mM NaCl, 3 mM KCl, 8 mM Na_2_HPO_4_, 1.5 mM KH_2_PO_4_ pH 7.4), retained proteins were separated by SDS-PAGE and stained with silver nitrate.

For western blot analysis, cells were counted after trypsinization to allow the loading on SDS-PAGE of about 10 µg total proteins corresponding to 100,000 cells. Detection of the EGFP-IN fusion protein was performed using a mouse monoclonal anti-EGFP antibody (Clontech) at a dilution of 1/1000 with overnight incubation. Binding of the primary antibody was revealed by a secondary HRP-conjugated anti-mouse antibody (Interchim, cat# 715 035 151) used at a dilution of 1/2000 in PBS with 0.1% BSA and incubated for 1 hour at room temperature. Anti-actin primary antibody from Sigma was also used as loading control at a dilution of 1/1000.

For microscopy experiments, cells were grown on a glass coverslip, transfected as described above for 24 h, fixed with 4% paraformaldehyde. Coverslips were mounted onto glass slides using VECTASHIELD Antifade Mounting Medium with DAPI (4′,6-Diamidino-2-Phenylindole, Dihydrochloride). Representative images were obtained using an AxioImager Z1 (Zeiss).Quantification of IN expression through the EGFP fluorescence was performed as described in the following “Transduction assay” section.

### Mammalian cells

Human 293 T cells were obtained from the ATCC while MEF cells knock-out (KO) for GCN2 (GCN2^−/−^) and their relative parental cells (GCN2^+/+^) were a kind gift from Dr. P. Fafournoux, (UMR1019, Clermont-Ferrand, France). Cells were maintained at 37 °C in a humidified atmosphere containing 5% CO_2_ in complete DMEM medium (containing 10% heat inactivated fetal calf serum) and supplemented with 55 µM β-mercaptoethanol for MEF cells.

### Viral production

Single-round vectors were produced by transfection of 293 T cells with three different plasmids for HIV-1 based lentiviral vectors [the WT or mutant packaging plasmid pCMVΔR8.91 (10 µg), the pCMV-VSV-G plasmid coding for the envelop (5 µg), and the pRRLsin-PGK-EGFP-WPRE reporter plasmid (15 µg)^25, 26^ and for the MLV-based retroviral vector (the reporter plasmid pRSF91.GFP.pre, the plasmid coding for the envelop pMD2.G and the packaging plasmid pHIT60). Cells (5 × 10^6^) were plated on 10-cm dishes and transfected the following day by calcium phosphate DNA precipitation. After 48 h, the viral supernatant was collected, filtered through 0.22-µm filters, concentrated by ultracentrifugation, treated with DNase I (Promega) at 40 U/ml for 1 h at 37 °C, and stored at −80 °C. Viral titration was determined by ELISA through p24 quantification (InGen - International Genetics Technologies).

### Real-time quantitative PCR

DNA was extracted from infected cells at 48 h post-infection using the QIAamp DNA Blood Mini kit (QIAGEN) and DNA concentration was adjusted to 20 ng/µl for each sample. A serial dilution of pRRLsin-PGK-EGFP-WPRE lentiviral plasmid was used to derive a standard curve. For total DNA quantification, qPCR reactions were performed using the KAPA SYBR® FAST qPCR kit to amplify the EGFP coding region. The reaction was performed with 5 µl of sample at a 1/10, 1/100 and 1/1000 dilution, 200 nM of primer Lenti F (GGAGCTAGAACGATTCGCAGTTA) and Lenti R (GGTTGTAGCTGTCCCAGTATTTGTC), 0.4 µl of ROX high and 10 µl mix KAPA 2X mix in a final volume of 20 µl. The PCR program was set to 95 °C for 3 minutes, 42 cycles (3″ at 95 °C, 20″ at 60 °C and 20″ at 72 °C). Integrated DNA was quantified using a one-step, real-time PCR amplification of the EGFP gene with iQTM SYBR® Green Supermix (Bio-Rad), 50 ng of total DNA, two primers (EGFP sense, 5′-CGCACCATCTTCTTCAAGG-3′, and EGFP antisense, 5′-GTGTCGCCCTCGAACTTCAC-3′) and an EGFP probe (5′-CGGCAACTACAAGACCC-3′). The amplification reaction was performed using a step at 50 °C for 2 minutes and a step at 95 °C for 10 minutes, followed by 40 cycles of 15″ at 95 °C and 1′ at 60 °C. Normalization was performed by comparison of the EGFP gene to the endogenous RNase P using the Ct method for each sample.

### Transduction assay

Cells were plated in 48-multiwell plates at 50,000 cells/well. For murine-based retroviruses, cells were transduced immediately after seeding as compared to on the following day with HIV-based vectors. After 48 hours, cells were washed twice with PBS, treated with trypsin, and then resuspended in 200 µl PBS, 200 mM EDTA, 0.5% FCS. Fluorescence was quantified using 10,000 cells on a FACS Calibur flow cytometer (Becton-Dickinson, San Jose, CA). Plots were analyzed using the FCS express v3.00.0103 software.

### Integration sites by deep sequencing

Determination of the integration sites was performed according to previous publication^27^. Total DNA was extracted after infection with the QIAamp DNA Blood Mini kit. DNA was digested with *Mse*I (New England Biolabs) overnight at 37 °C. *Mse*I linkers were ligated to the DNA overnight at 21 °C. Digestion with *Dpn*I and *Sac*I was performed for 4 h at 37 °C to eliminate starting DNA and internal junction LTR-viral DNA. Amplification was performed with Phusion High Fidelity DNA Polymerase in the presence of 300 nM of LTR1 and MseL1 primers (CTTAAGCCTCAATAAAGCTTGCCTTGAG and GTAATACGACTCACTATAGGGC, respectively), 250 nM dNTPs, 5 µl HF 5X buffer and 3 mM MgCl_2_. The amplification program consisted in a denaturation step at 95 °C for 2 minutes followed by 25 cycles (15″ at 95 °C, 30″ at 55 °C and 1′ at 72 °C). After a 1/50 dilution, the PCR product was subsequently used as a template for a nested PCR using primers MseL2 (CGTATCGCCTCCCTCGCGCGCCATCAGAGGGCTCCGCTTAAGGGAC) and LTR2 454 mid (CTATGCGCCTTGCCAGCCCGCTCAGC-N10-AGACCCTTTTAGTCAGTGTGGAAAATC, with N_10_ corresponding to the Mid sequences). The resulting DNA products were ethanol precipitated overnight, the pellet was resuspended in 20 µl crystal violet loading dye 6X [kit S.N.A.P UV-Free Gel Purification (Invitrogen)] and loaded on a 2% agarose gel in TAE buffer stained with crystal violet. DNA fragments with a length of 200 to 500 base pairs (bp) were extracted and sequenced using the 454 Junior sequencer (Roche).

### Integration sites analysis

Reads from nested PCR that matched against the primer sequence were cleaned using an in-house Perl script (available on request) that removes both, PCR primers and viral sequences. Resulting sequences correspond to the genomic portion of the host localized immediately after the 3′-LTR. Sequences of 50 bp in length and above were then aligned against the reference human genome (hg38) using BLAT (Version 34)^28^. To be included in the analysis, BLAT output sequences must be aligned against only once on the host genome (unique integration site) with a score of at least 98%. To account for a potential bias due to the use of the MseI restriction enzyme during sample preparation, we generated theoretical integration sites using in-house Perl scripts (match random control, MRC) that play the role of control sets, similarly to previous publication^28^. These theoretical sites were generated according to two criteria: (i) they have to be randomly distributed along the genome and (ii) they must be located at a distance equal to the distance between the restriction enzyme site and the related experimental site^29^. For each identified unique site, 10 *in silico* MRC were generated. Correlation between the integration sites and various genomic features (*e*.*g*. gene body, histone modifications) was tested using statistical analysis previously described in ref. 28. Experimental data was then compared to the *in silico* generated MRC set using a chi-square test.

## Results

In a previous study, we identified GCN2 as a potential cellular partner of HIV-1 IN using the two-hybrid approach^15^. Later on, we confirmed that human GCN2 interacts with HIV-1 IN, as observed by co-immunoprecipitation, and that GCN2 could act as a restriction factor as silencing of GCN2 by siRNA increased viral replication^8^. To investigate the mechanism by which this cellular protein kinase can affect HIV-1 replication, we first conducted *in vitro* phosphorylation experiments to determine the impact of HIV-1 IN on GCN2 activity and whether IN could be phosphorylated by GCN2.

### HIV-1 IN is a substrate for GCN2 without competing with eIF2α’s phosphorylation

**Figure 2:**
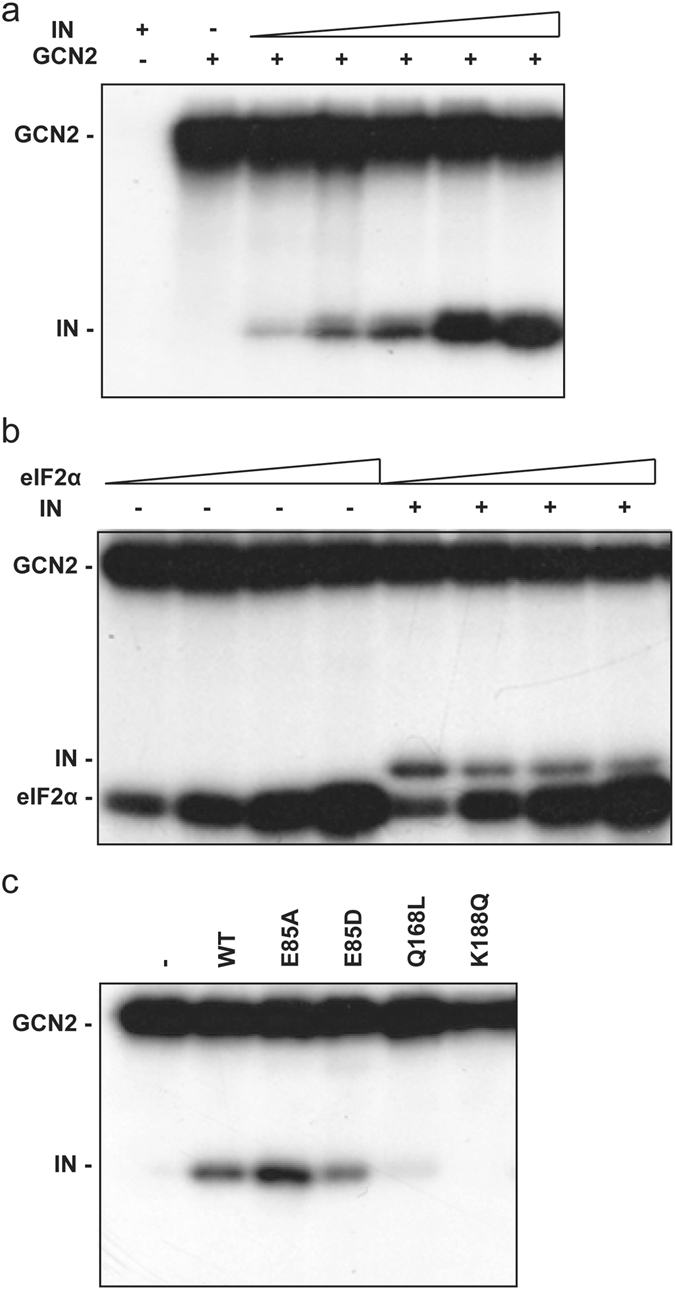
GCN2 *in vitro* phosphorylation assay. (**a**) Phosphorylation of HIV-1 IN catalyzed by GCN2. Lane 1, HIV-1 IN (2000 nM) alone. Lane 2 to 7, GCN2 (24 nM) was incubated with [γ-^32^P]-ATP and increasing concentrations of HIV-1 IN (0, 100, 225, 450, 1000, 2000 nM). (**b**) Competition assay. GCN2 phosphorylation of increasing concentrations of eIF2α (from 100 nM to 800 nM following a 2-fold increment) with radiolabeled [γ-^32^P]-ATP, in the presence or not of HIV-1 IN (2 µM). (**c**) Impact of HIV-1 IN oligomerization on phosphorylation by GCN2. Lane 1, no IN. Lanes 2 to 6, HIV-1 IN WT or mutants (700 nM).

For in *vitro* phosphorylation experiments, HIV-1 IN was expressed in bacteria to ascertain the absence of pre-existing phosphorylation (verified by mass spectrometry, data not shown). GCN2 incubated with [γ-^32^P]-ATP did undergo autophosphorylation and a signal could be observed only at the protein size corresponding to GCN2 (Fig. 2a, lane 2). Then, purified GCN2 was incubated in the presence of increasing concentrations of IN. In addition to GCN2 autophosphorylation signal, a proportional radiolabeling was observed at the protein size corresponding to IN (Fig. 2a). This radiolabeling was specifically associated with the presence of GCN2 as in the absence of the kinase no phosphorylation could be detected (Fig. 2a, lane 1). Thus, it appeared that GCN2 was able to phosphorylate HIV-1 IN.

Next, we determined whether IN could interfere with GCN2’s physiological activity (*e*.*g*. phosphorylation of eIF2α). Using increasing concentrations of recombinant eIF2α also led to a proportional labeling of the protein in the presence of GCN2 (Fig. 2b, lanes 1 to 4). Then, adding an excess of IN (2 µM), did not decrease the apparent phosphorylation level of eIF2α (Fig. 2b, lane 5, compare lane 5 to lane 1). Furthermore, increasing amount of eIF2α resulted in a slight decrease in HIV-1 IN radiolabeling (Fig. 2b, lanes 5 to 8). Thus, HIV-1 IN is a new substrate for GCN2 that does not compete with eIF2α’s phosphorylation.

HIV-1 IN residue E_85_ was shown to participate in the enzyme oligomerization and more precisely to form a salt-bridge at the dimer interface (at the interface of one structural and one catalytic subunit)^30^. E85D is a conservative mutation that should not drastically impact the capability of IN to form this salt-bridge interaction. On the opposite, E85A should abolish this interaction and increase the hydrophobic property of this region. Under native conditions (BN-PAGE), mutation E85A increased the proportion of high molecular weight complexes of IN, thus favoring oligomerization/aggregation (data not shown). In the phosphorylation assay, the E85D mutant was less phosphorylated compared to WT whereas the E85A mutant showed an increased level of phosphorylation compared to the E85D or the WT IN (Fig. 2c). To confirm a potential effect of IN oligomerization, other mutations previously shown to impair IN multimerization (*i*.*e*. exclusively forming monomers in solution) such as the Q168L and K188Q were used in the phosphorylation assay^31^. Neither the Q168L nor the K188Q mutant were an efficient substrate for GCN2 (Fig. 2c, lanes 5 and 6, respectively). These results are consistent with the hypothesis of a specific phosphorylation of IN oligomers by GCN2.

### Identification of HIV-1 IN residues phosphorylated by GCN2

To determine which IN residues were phosphorylated by GCN2 *in vitro*, IN was incubated with or without GCN2 in the presence of unlabeled ATP. In the absence of GCN2, IN expressed in bacteria did not present any detectable phosphorylated residue as determined by LC-MS/MS. On the other hand, two phosphorylation sites were unambiguously identified in the presence of GCN2, serine residues at positions 24 and 255 of IN (Supplemental Table S1). These residues were found highly conserved among 590 HIV-1 IN sequences obtained from infected patients (conserved at 87% and 93% at position 24 and 255, respectively, data not shown). Interestingly, HIV-1 IN residue S_57_ that was recently shown to be phosphorylated by JNK^6^ was not phosphorylated by GCN2 in our conditions.

**Figure 3:**
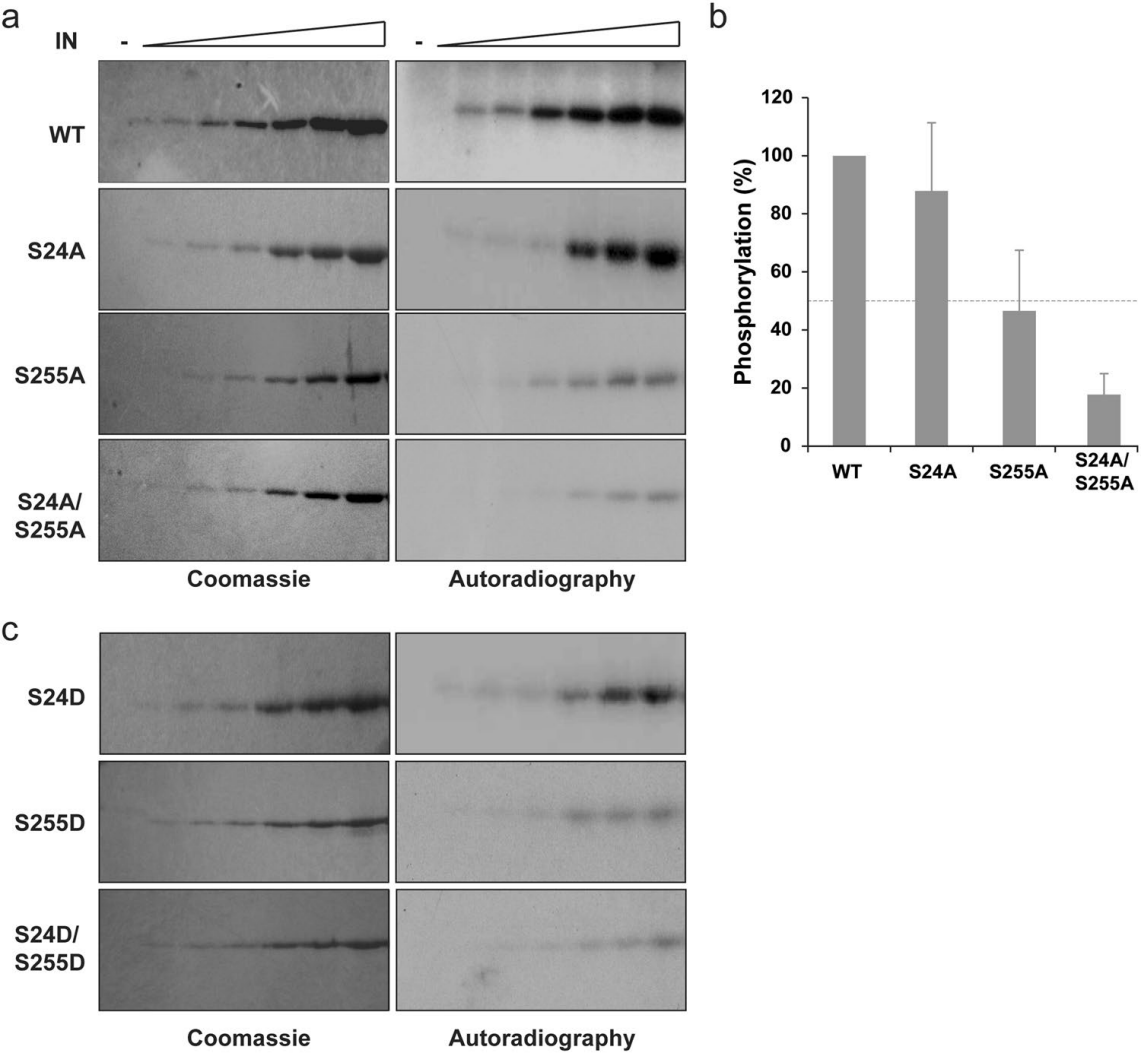
Phosphorylation of phosphomutant INs. Phosphorylation of recombinant WT IN compared to alanine (**a**) and aspartic acid (**c**) mutant. Increasing concentrations of IN (0, 100, 200, 400, 1000, 2000, 4000 nM) and 24 nM of GCN2 were used. Gels staining with Coomassie blue (left) and autoradiography (right). Quantification at the highest phosphorylation level for each protein (**b**). Mean and standard deviation derived from at least three independent experiments and were normalized to WT.

To confirm the role of residues S24 and S255 in the phosphorylation process, point mutations were introduced in the IN protein. A serine to alanine mutation can be used to block a phosphorylation site. On the opposite, a serine to aspartic acid or glutamic acid can be used to mimic a constitutive phosphorylation. Thus, six “phosphomutants” were generated: four INs with a single mutation (IN S24A, IN S24D, IN S255A, and IN S255D), and two with double mutations (IN S24A/S255A and IN S24D/S255D). As expected, mutating simultaneously the two identified sites to alanine (S24A/S255A) resulted in a weak level of phosphorylation catalyzed by GCN2 (Fig. 3a). Thus, IN residues S24 and S255 were the main phosphorylation sites targeted by GCN2. While mutation S255A strongly decreased the phosphorylation of the protein, alanine mutation at position 24 had less impact on IN’s phosphorylation. When comparing the phosphorylation level obtained at 2 µM of IN, the S24A was phosphorylated at about 90% of the levels of the WT (Fig. 3b) while the phosphorylation of the S255A mutant was strongly decreased (around 50% of WT). Mutating both the S24 and S255 to alanine resulted in almost complete abolition of phosphorylation (20% of the WT phosphorylation level, Fig. 3b). Next, phosphorylation of the S24D and S255D mutants were analyzed (Fig. 3c). The double mutant S24D/S255D severely impaired the phosphorylation catalyzed by GCN2 and the S255D mutation affected more drastically this phosphorylation of IN than mutation S24D. Altogether, these results indicated that the absence of a serine residue at position 255 prevented efficient phosphorylation of IN, which suggests that IN residue S255 is the primary phosphorylation site targeted by GCN2 *in vitro*.

**Figure 4:**
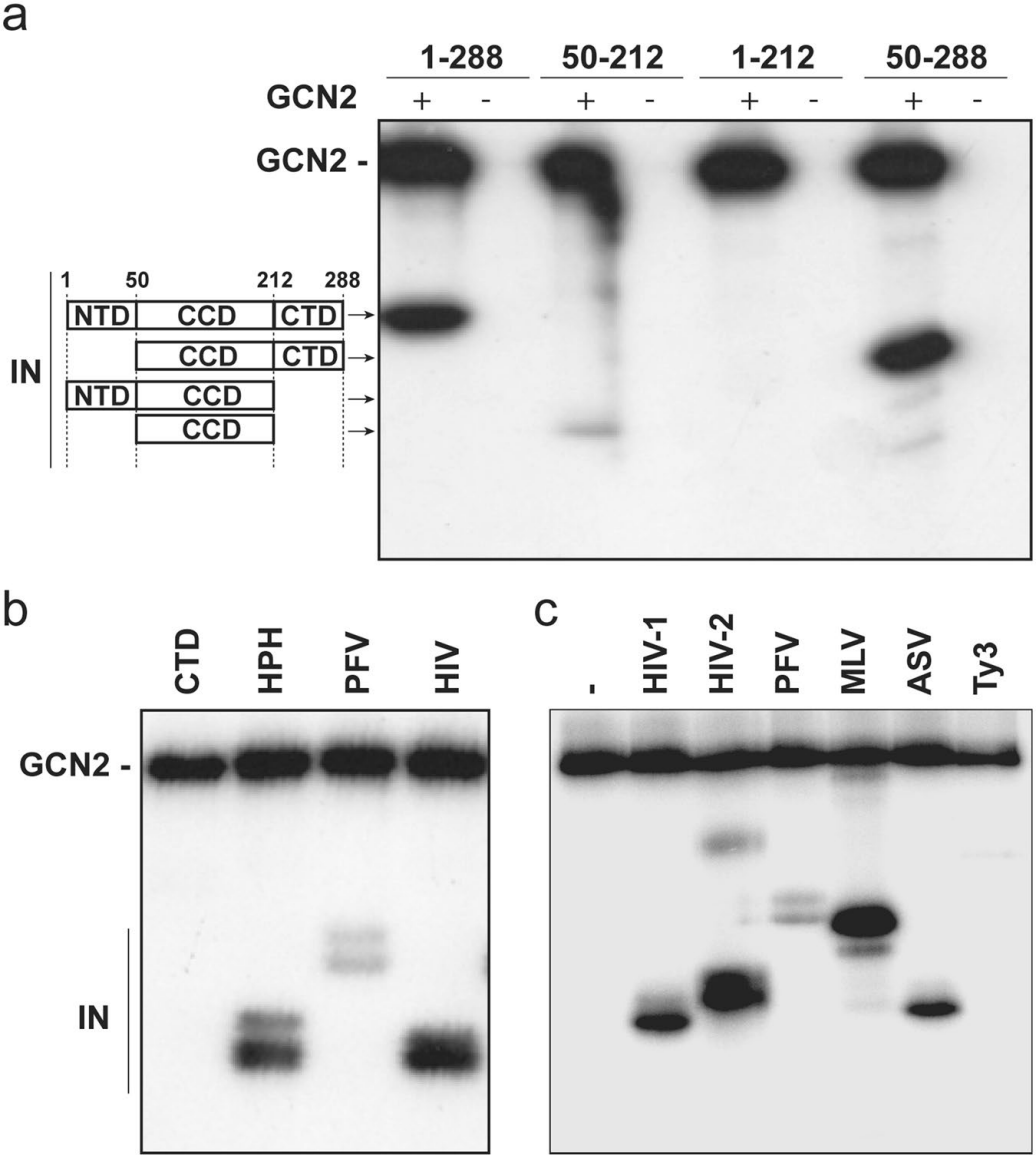
Determination of the IN domains targeted by GCN2. (**a**) Phosphorylation by GCN2 of the full-length HIV-1 IN (1–288), the CCD (50–212) or the two-domain fragments NTD+CCD (1–212) and CCD+CTD (50–288). (**b**) Phosphorylation by GCN2 of the isolated HIV-1 CTD (212–288), the HPH chimeric protein and the IN from PFV and HIV-1. (**c**) Phosphorylation by GCN2 of full-length IN.

To confirm this major contribution of S255 in the phosphorylation of the IN, the different truncated IN-proteins CCD (50–212), NTD-CCD (1–212) and CCD-CTD (50–288) were analyzed in a phosphorylation assay. Compared to the full-length IN, only close to background signal was observed at the size of the isolated CCD domain (Fig. 4a, compare lane 1 and 3). Using non-radioactive ATP and mass spectrometry, multiple phosphorylations were detected at different positions of the IN catalytic core but lacked significant reproducibility (data not shown). Similarly, GCN2 was not able to phosphorylate the two-domain fragment 1–212 containing both the NTD and CCD (Fig. 4a, lane 5). However, mass spectrometry enabled the reliable detection of a phosphorylation at IN position 24 (Supplemental Table S1). This apparent discrepancy can be explained by a low level phosphorylation under these experimental conditions detected thanks to the higher sensitivity of the biophysics approach compared to the gel-based assay. Finally, the 50–288 fragment including the CCD and CTD was efficiently phosphorylated (Fig. 4a, lane 7) and a unique phosphorylation site at position 255 was identified by mass spectrometry analysis (Supplemental Table S1). Surprisingly, no radiolabeling was observed when the phosphorylation assay was conducted with the isolated CTD (Fig. 4b, lane 1), meaning that the IN CTD is not a suitable substrate for GCN2 in the absence of the CCD. Altogether, these results confirm that the CTD contains the main site of phosphorylation by GCN2 and that the CCD contains crucial determinants for GCN2 recognition.

To better understand the role of the IN CCD, a chimeric protein (HPH) was used that harbors the HIV-1 IN NTD (residues 1–56) fused to the PFV IN CCD (residues 116–278 corresponding to HIV-1 IN residues 57–209)^19^ and the HIV-1 IN CTD (residues 221–288). In this context, GCN2 was able to phosphorylate the chimeric protein (Fig. 4b, lane 2). Thus, the required features for efficient phosphorylation by GCN2 are common in the IN CCD of both PFV and HIV-1. However, GCN2 was unable to efficiently phosphorylate the native PFV IN (Fig. 4b, lane 3) indicating that the CTD of PFV IN is missing an important determinant present in HIV-1 IN.

Because PFV IN is somewhat divergent from HIV-1 IN, out of the highly conserved active site, recombinant IN from various other viruses were used to investigate the role of the CTD in the phosphorylation process (Fig. 4c). Unsurprisingly, GCN2 was able to efficiently phosphorylate IN from HIV-2, the closest virus to HIV-1 (Fig. 4c, lane 3). Moreover, GCN2 was also able to phosphorylate IN from MLV and ASV (Fig. 4c, lanes 5 and 6, respectively). On the other hand, a close to background signal was observed when using the IN from the retrotransposon Ty3 as substrate for GCN2, thus even weaker than that observed with the IN from PFV (Fig. 4c, lanes 7 and 4, respectively). Altogether, phosphorylation of IN by GCN2 seemed to be dependent on features of the CCD that are common to all retroviruses and features of the CTD that are restricted to viruses of the *Orthoretrovirinae* subfamily.

### Residues S24 and S255 of HIV-1 IN are also phosphorylated *in cellulo*

Next, we determined whether HIV-1 IN could be phosphorylated in a cellular context. First, viral proteins were extracted from native HIV-1 particles and submitted to mass spectrometry. Within the mature virion, we could not detect any phosphorylation of the IN protein (data not shown). Because the interaction of IN with GCN2 was initially uncovered during a yeast two-hybrid assay, we first investigated whether this interaction could led to an effective phosphorylation of IN in the yeast cell. HIV-1 IN expressed in *S*. *cerevisiae* and purified in a manner similar to that of the protein expressed in bacteria was phosphorylated at the same residues targeted by GCN2 *in vitro*, namely S24 and S255 (data not shown). Thus in yeast, GCN2 interacted with IN and recognized it as a substrate leading to its phosphorylation. Moreover, phosphorylation of residues S24 and S255 was also detected by mass spectrometry when IN, fused to EGFP, was over-expressed in 293 T cells (Supplemental Table S1). Altogether, these experiments indicated that *in vitro* GCN2 was capable of phosphorylating two serine residues of HIV-1 IN that were also phosphorylated in a cellular context. Thus, GCN2 constitutes a good candidate for the phosphorylation of HIV-1 IN in human cells.

### Effect of phosphomutants on HIV-1 replication

**Figure 5:**
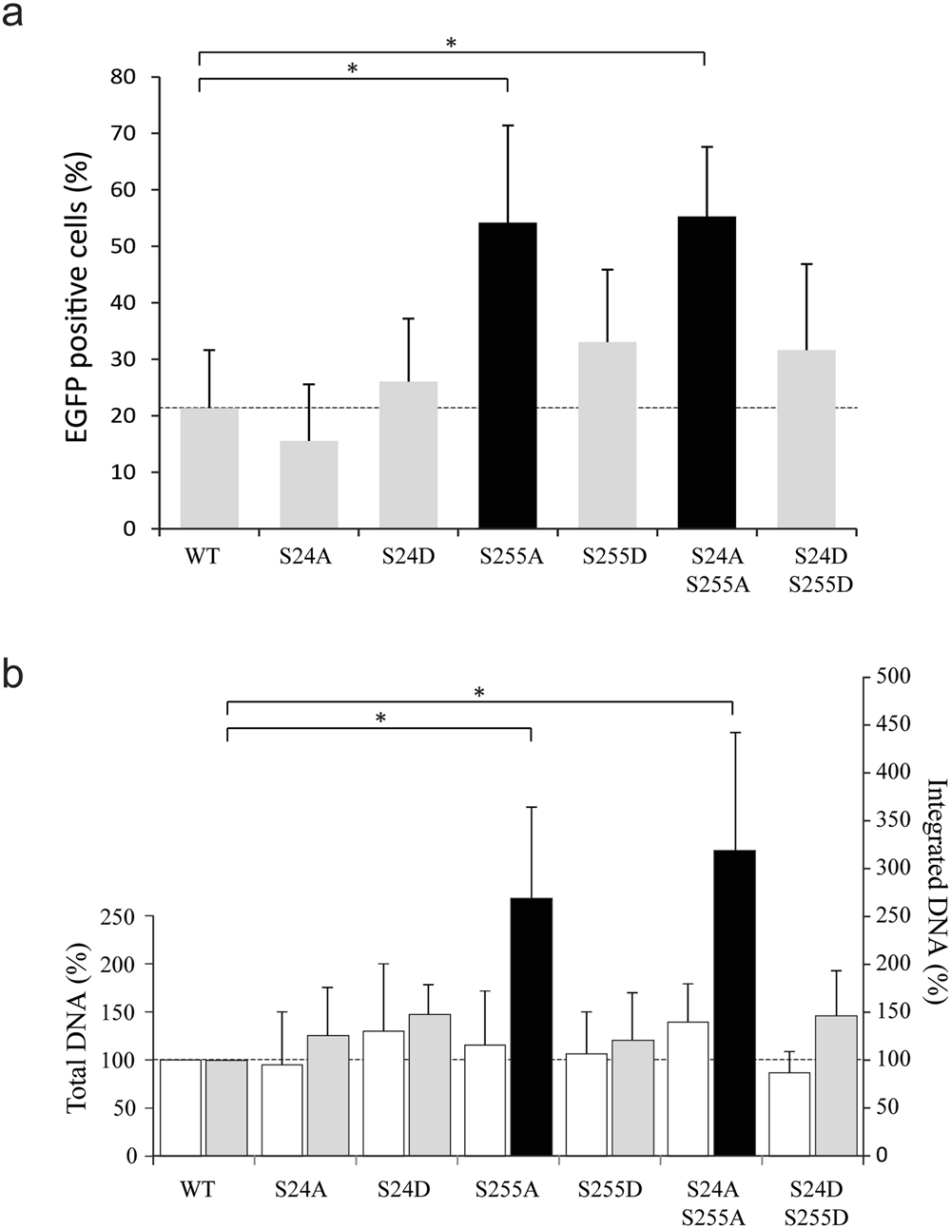
Effect of mutations on infectivity. Transduction efficiency of 293 T cells using WT and mutant vectors at a MOI of 1 was determined by measure of EGFP expression by flow cytometry (**a**) and by qPCR to quantify total and integrated viral DNA (**b**). The results derived from the mean of at least three independent experiments. Student’s *t*-test *p < 0.05.

To evaluate the effect of IN phosphorylation during viral replication, lentiviral particles carrying mutated IN at residues S24 and S255 were produced. Infectivity of mutant vectors was then compared to the WT control by transducing cells with equivalent amounts of p24 and by measuring the EGFP expression by flow cytometry. Transduction with the WT vector led to around 20% of the cells detected as EGFP positive (Fig. 5a). Interestingly, mutating residue S24 and/or S255 to aspartic acid did not significantly alter the percentage of infected cells by the corresponding viruses. Thus, mimicking a constitutive phosphorylation of IN had no detectable impact on viral infectivity compared to the native condition. Besides, particles carrying the IN mutation S24A also transduced 293 T cells with a similar efficiency of 15%, (Fig. 5a). On the other hand, lentiviral vectors carrying the S255A IN mutation, alone or in combination with S24A (S24A/S255A), exhibited an infectivity significantly higher than that of the WT viruses (reaching around 55% of EGFP positive cells *versus* 20%). Hence, preventing phosphorylation at IN residue S255 enhanced infectivity of the corresponding virus with no to little impact of the phosphorylation status of S24. Because depletion of GCN2 in cells induced a similar increase in HIV-1 replication^8^, it seems that GCN2 might be the sole cellular factor involved in the phosphorylation of IN residues S24 and S255 during HIV-1 replication leading to viral restriction.

Because mutations of IN can have pleiotropic effects^32^, the different populations of viral nucleic acids were quantified. No significant differences in the amount of total viral cDNA were observed between the WT control and the mutant viruses (Fig. 5b). When looking at the level of integrated DNA, mutants that did not affect infectivity as measured by flow cytometry (*i*.*e*. S24A, S24D, S255D and S24D/S255D, Fig. 5a) had no significant impact on the integration rate (Fig. 5b). Conversely, cells transduced with viruses carrying the IN mutation S255A and S24A/S255A presented an approximate 3-fold increase in the number of integration events (Fig. 5b), which is in agreement with the observed increase in infectivity. Altogether, these results suggest that IN mutation S255A does not affect the entry and reverse transcription steps but likely interferes at the integration level.

### Effect of phosphomutants on IN cellular expression and localization

**Figure 6:**
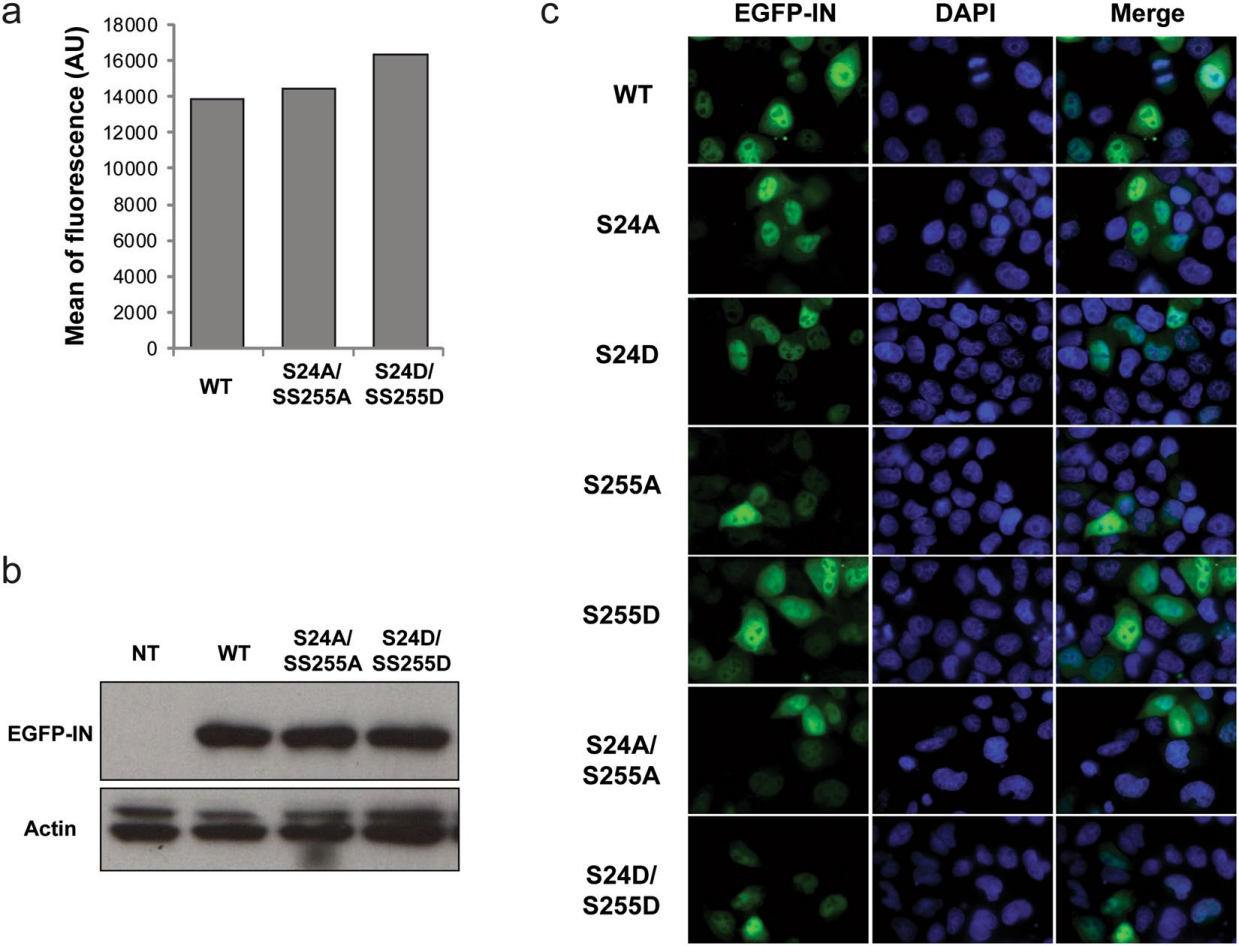
Effect of phosphorylation sites’ mutation on EGFP-IN expression in cells. 293 T transfected with a plasmid coding for WT or mutant EGFP-IN were analyzed by flow cytometry (**a**), western blot analysis (**b**) or microscopy (**c**). Lane 1: non transfected cells; lane 2: WT EGFP-IN; lane 3: S24A/S255A EGFP-IN; lane 4: S24D/S255D EGFP-IN. For microscopy, nuclei were stained with DAPI.

Phosphorylation is a versatile post-translational modification that plays crucial roles in the cell and particularly in the regulation of protein-protein interactions leading to the control of enzymes activity, stability and sub-cellular localization. Accordingly, WT or mutant INs were expressed as a fusion protein with EGFP in 293 T cells. As determined by flow cytometry, the mean fluorescence was similar in the context of WT IN or of the double mutant S24A/S255A or S24D/S255D (Fig. 6a). Moreover, the integrity of the fusion protein was evaluated by western blot using either an anti-IN antibody, or an anti-EGFP antibody (data not shown). A single band corresponding to the full-length fusion protein could be detected for the WT and mutated enzymes (Fig. 6b). Altogether, these results indicate that mutations of IN residues S24 and S255 did not significantly change the stability nor the expression of the fusion protein during the course of the experiment.

Because IN and the pre-integration complex need to be actively transported to the nucleus to enable viral integration into the host genome, whether IN phosphorylation could affect its cellular localization was investigated. As expected, the over-expressed WT EGFP-IN was enriched in the nucleus of transfected 293 T cells (Fig. 6c). A similar localization was observed for all of IN variants, whatever the nature of mutation (alanine or aspartic acid) and regardless of the position (S24, S255 or both). Thus, it seemed that phosphorylation of IN interfered with the integration efficiency without affecting the nuclear import of IN.

### Effect of phosphomutants on integration site selection

**Figure 7:**
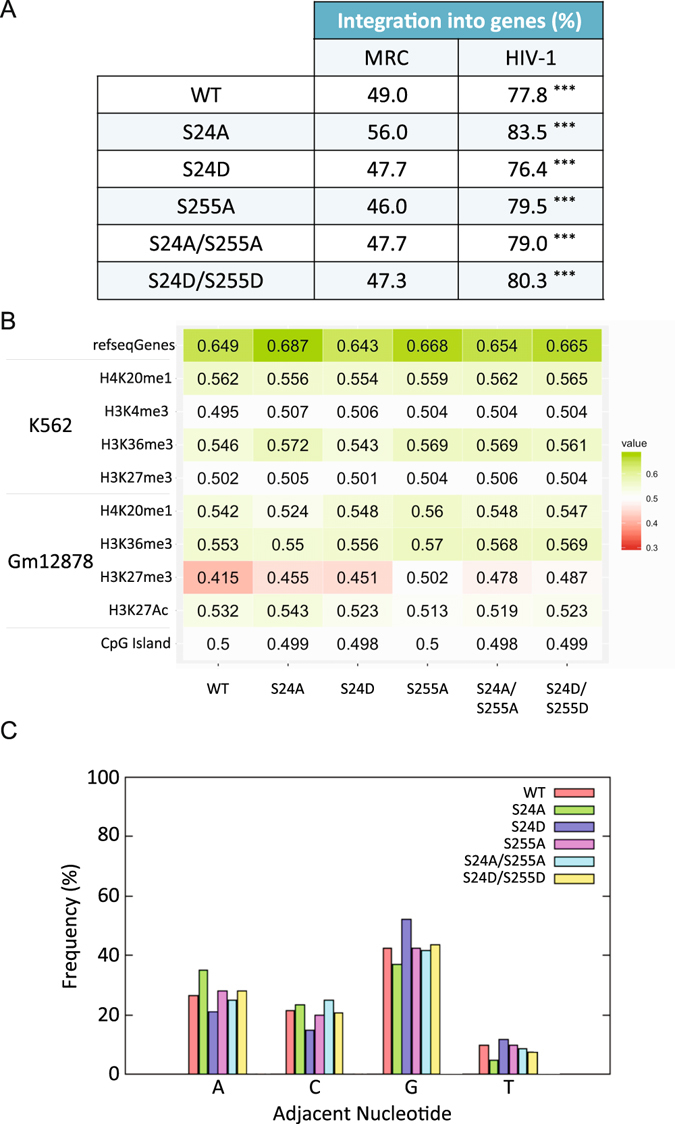
Effect of phosphomutants on integration site selection and specificity. (**a**) Frequency of integration into genes. Stars represent the p-values (***p < 0.001) after comparison of experimental results with matched random control. (**b**) Heatmap of integration site datasets. For a given integration site dataset, all calculated rank values are averaged. Values between 0 and 0.5: the genomic feature appears less frequently at/near integration sites than at/near random sites in the genome and is disfavored. Values between 0.5 and 1: the genomic feature is enriched at integration sites. Values of exactly 0.5 indicate that integration sites are neither enriched nor depleted with respect to the feature of interest. (**c**) Frequency of occurrence of each nucleotide at the integration site.

Integration of HIV-1 is known to occur into the body of genes^33^. To determine whether phosphorylation of IN might affect integration through a change in site selection and selectivity, ultra-deep sequencing of integration events arising from the transduction of WT or mutant lentiviral vectors was performed. First, the frequency of integration into the body of genes was determined. As expected, the WT virus was found to integrate mainly in active transcription units (Fig. 7a). The same specificity of integration was found for all the phosphomutants, with integration into genes representing approximately 80% of the analyzed sequences (Fig. 7a). This correlates with ROC area obtained with refseqgenes as feature showing that integration occurred preferentially in the body of genes for the WT and the various mutants (Fig. 7b). On the opposite, there was no specific enrichment of integration sites in CpG islands compared to MRC. We were unable to generate enough data with the S255D single mutant to be statistically significant. Consequently, this single mutant was excluded from the analysis to the benefit of the S24D/S255D double mutant.

Next, the integration sites were compared to the position of histone modifications. Because there is no precise map of histone modifications in 293 T cells, already published data obtained in two different cell lines, namely in K562 and Gm12878 cells were used. Position of the integration sites sequenced in 293 T correlated positively with regions presenting H4K20me_1_ and H3K36me_3_ in both cells lines (Fig. 7b). Both histone modifications are highly associated with transcribed genes and for the latter also linked to LEDGF/p75, the IN tethering factor. Moreover, integration sites of the WT vector appeared disfavored in regions with H3K27me_3_ (silenced chromatin) in Gm12878 cells and at least not favored in K562 cells. The same result was obtained with all our IN mutants except for the S255A which wasn’t disfavored in Gm12878 cells (Fig. 7b). These results show that mutations at IN residue S24 and S255 did not change the selectivity for specific genomic features or histone modifications at the integration site.

Finally, the nature of the nucleotide adjacent to the integration site was examined and the frequency for each of the four bases was calculated. In the context of the WT virus, integration preferentially occurred next to a G nucleotide with a prevalence of around 40% (Fig. 7c). Moreover, integration was strongly disfavored next to a T nucleotide with a prevalence of about 10%. Finally, the prevalences of A and C nucleotides were similar at around 25% and 20%, respectively. These values are in agreement with data from the literature^34^. Transducing cells with viruses carrying IN mutations at position S24 or S255 did not affect this profile and prevalence of each nucleotide at the integration site was not significantly different. Thus, we conclude that mutation S255A increased the integration efficiency of IN without affecting the selectivity of integration.

### Effect of serine 24 and 255 mutations on IN catalysis *in vitro*

**Figure 8:**
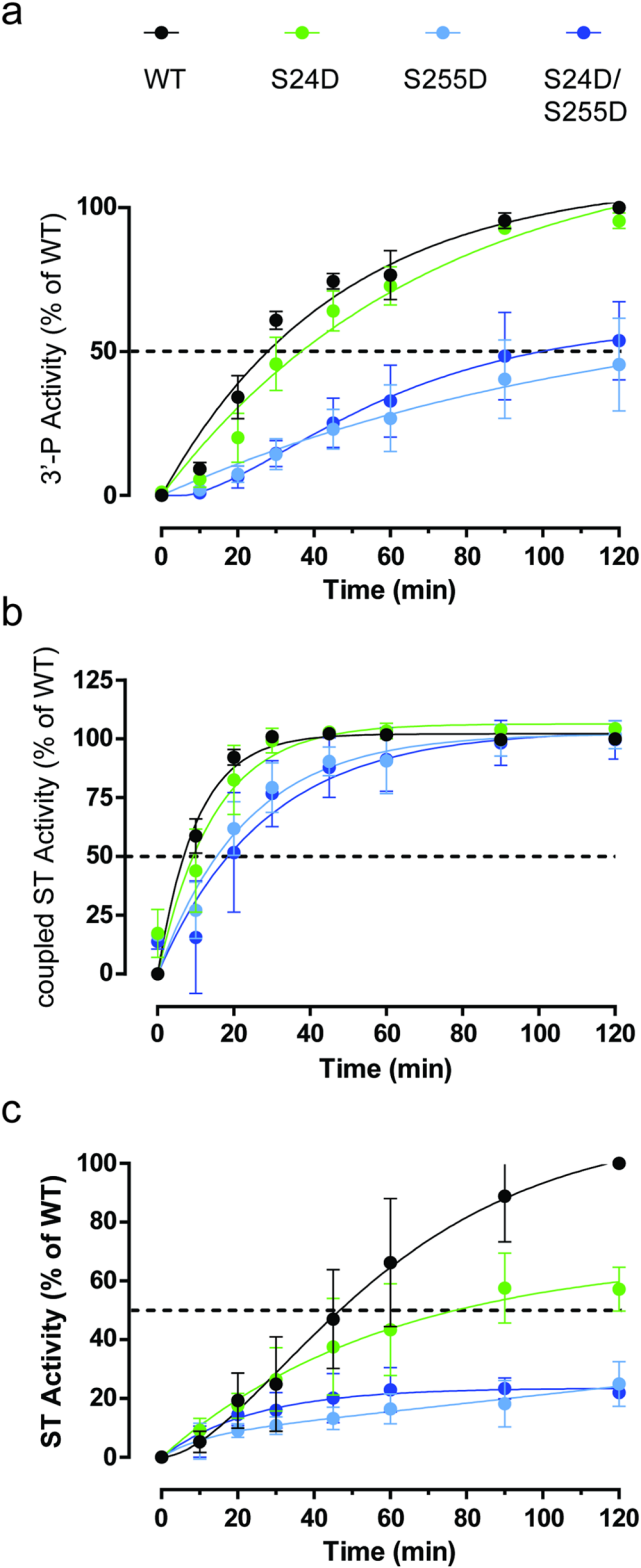
Effect of mutations on HIV-1 IN *in vitro* activity. Time course experiments were performed to monitor the 3′-P activity (**a**), the resulting ST activity (coupled ST, panel b) and the ST independently of 3′-P (uncoupled ST, panel c). After separation on acrylamide gel and autoradiography, the products were quantified and normalized to the level of the WT enzyme activity.

To determine whether phosphorylation of HIV-1 IN could modulate its enzymatic activities, the *in vitro* 3′-end processing, strand transfer and concerted integration activities of the six recombinant mutants were compared to the WT enzyme (Fig. 8). In agreement with infectivity data, the 3′-P activity of the S24D mutant was comparable to that of the WT enzyme (Fig. 8a). Mutants harboring the S255D variation, with or without the S24D mutation, exhibited a 2-fold decrease in 3′-P activity (Fig. 8a). After processing the DNA, IN is able to carry on and to catalyze the strand transfer (ST) of this 3′-P product into other DNA molecules. Quantification of ST products originating from the 3′-P reaction showed that even if the S255D and S24D/S255D mutants were delayed in ST catalysis the end-point level of catalysis was similar to the WT enzyme (Fig. 8b).

Then, a 19/21 pre-cleaved substrate was used to monitor the ST reaction independently of the 3′-P. In this case, the S24D single mutant was able to catalyze ST at about 60% of WT level while the S255D and S24D/S255D mutants were profoundly affected with only 25% of the WT level (Fig. 8c). The apparent discrepancy between these results can be explained by a defect in DNA binding. In the ST reaction coupled to 3′-P, all of the 19/21 product is already bound to IN while, in the uncoupled ST reaction, IN first need to bind its pre-cleaved DNA substrate. This is in agreement with the fact that the S255D mutation introduced additional negative charges that might inherently decrease DNA affinity. However, it remains unclear why a similar effect was not observed in the case of the S24D mutation. Nevertheless, this trend is in agreement with cellular data showing that viruses mimicking a constitutive phosphorylation at IN position S255 (S255D and S24D/S255D) had a lower transduction efficiency and integration rate than viruses with a S255A mutation unable to sustain phosphorylation (Fig. 5). Altogether, it seems that phosphorylation of IN at position S255 might regulate its enzymatic activity.

### GCN2 decreases the replication of multiple retroviruses

**Figure 9:**
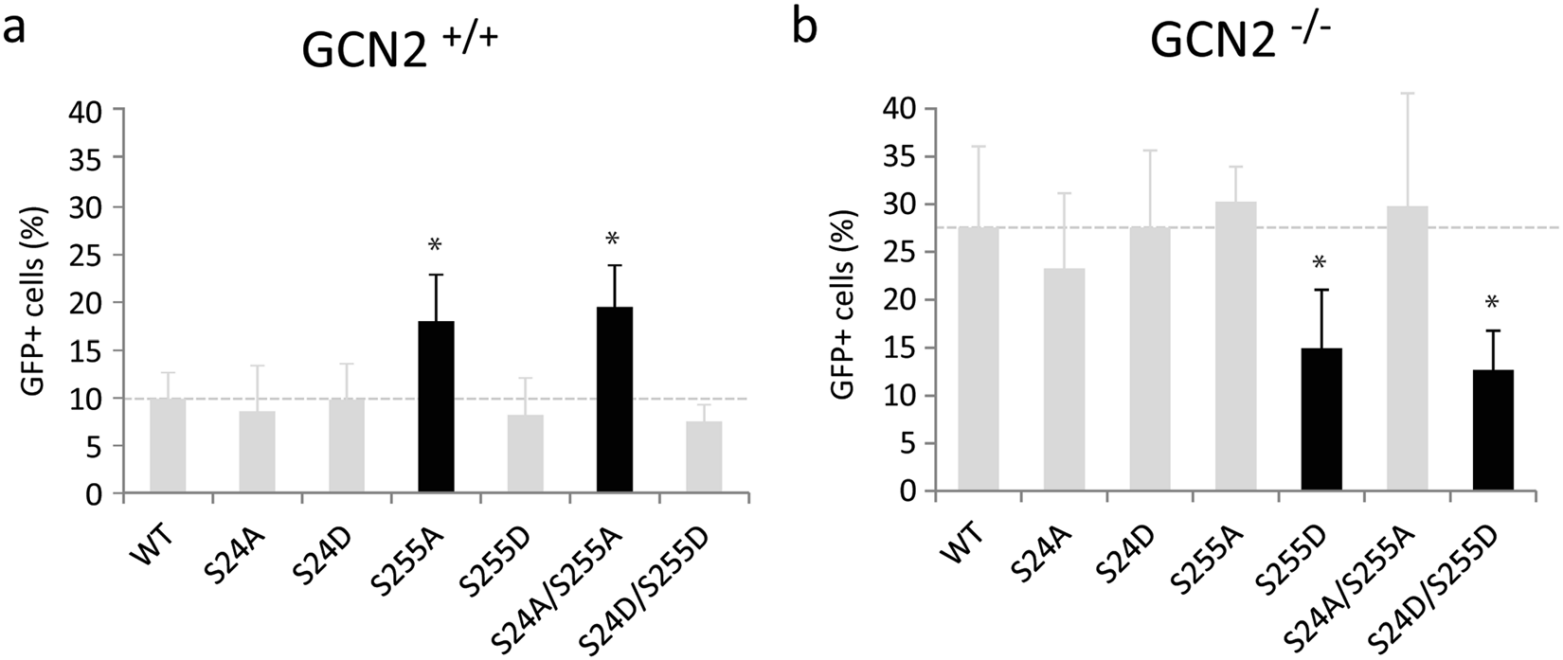
Infectivity of HIV-1-based vector in GCN2^−/−^ and GCN2^+/+^ MEF cells. MEF cells WT (GCN2^+/+^) (**a**) or knock out for GCN2 (GCN2^−/−^) (**b**) were transduced with a M.O.I. of 1 for 48 hours. The percentage of transduced cells was determined by flow cytometry. The data are the results of at least three independent experiments. Student’s *t*-test *p < 0.05.

GCN2 was able to phosphorylate IN from HIV-1 but also from other retroviruses such as MLV (Fig. 4c). To address the question whether GCN2 could restrict viral infections other than HIV-1, knockout MEF cells for GCN2 (GCN2^−/−^) were used. Utilization of GCN2^−/−^ and of a WT parental line as control (GCN2^+/+^) recapitulated the effect of siRNA knockdown during HIV-1 infection (Fig. 9). Transduction efficiency of a WT HIV-1 vector was significantly increased in GCN2^−/−^ MEF cells, compared to GCN2^+/+^ cells (27.6% of GFP positive cells *versus* 9.9%). In addition, sequencing of the integration sites obtained in these two cell lines revealed a similar pattern with integration occurring preferentially in the body of genes (data not shown).

In parallel, the infectivity of HIV-1 vectors harboring mutations at the IN phosphorylation site in the context of GCN2^+/+^ MEF cells was tested. In this cellular context, vectors carrying the aspartic acid mutations (S24D, S255D and S24D/S255D) infected the cells similarly to the WT vector with 9.8, 8.2 and 7.5% of EGFP positive cells, respectively. Conversely, an increase in transduction efficiency with viruses harboring the S255A or S24A/S255A IN mutations (Fig. 9a, left panel) was observed. The proportion of EGFP positive cells was 18 and 19.5% respectively, which is close to the 27.6% observed with the WT vector in the GCN2^−/−^ cells. This is in agreement with the results obtained using 293 T cells (Fig. 5a) and validates this mouse model.

Interestingly, using GCN2^−/−^ MEF cells led to a diametrically opposite results. In this case, infectivity of mutant vectors carrying IN mutation S255A or S24A/S255A remained comparable to that of the WT lentiviral vector (30.2% and 29.8%, respectively), (Fig. 9b, right panel). But, infectivity of viruses carrying the IN mutations S255D and S24D/S255D was decreased in GCN2^−/−^ MEFs compared to the WT virus. The proportion of EGFP positive cells was 15% and 12.7% respectively, compared to 27.5% for the WT virus in the same cell line. In agreement with this result, the infectivity of the S255D and S24D/S255D vectors in the GCN2^−/−^ cells was comparable to the infectivity of the WT vector in the GCN2^+/+^ cells. Thus, in the presence of GCN2, preventing IN phosphorylation with the S255A mutation increased the infectivity at a level similar to that observed in a cell lacking GCN2. Conversely, mimicking a phosphorylation with the S255D mutation in the context of GCN2^−/−^ cells decreased the infectivity at a level similar to the WT virus in the presence of GCN2.

**Figure 10:**
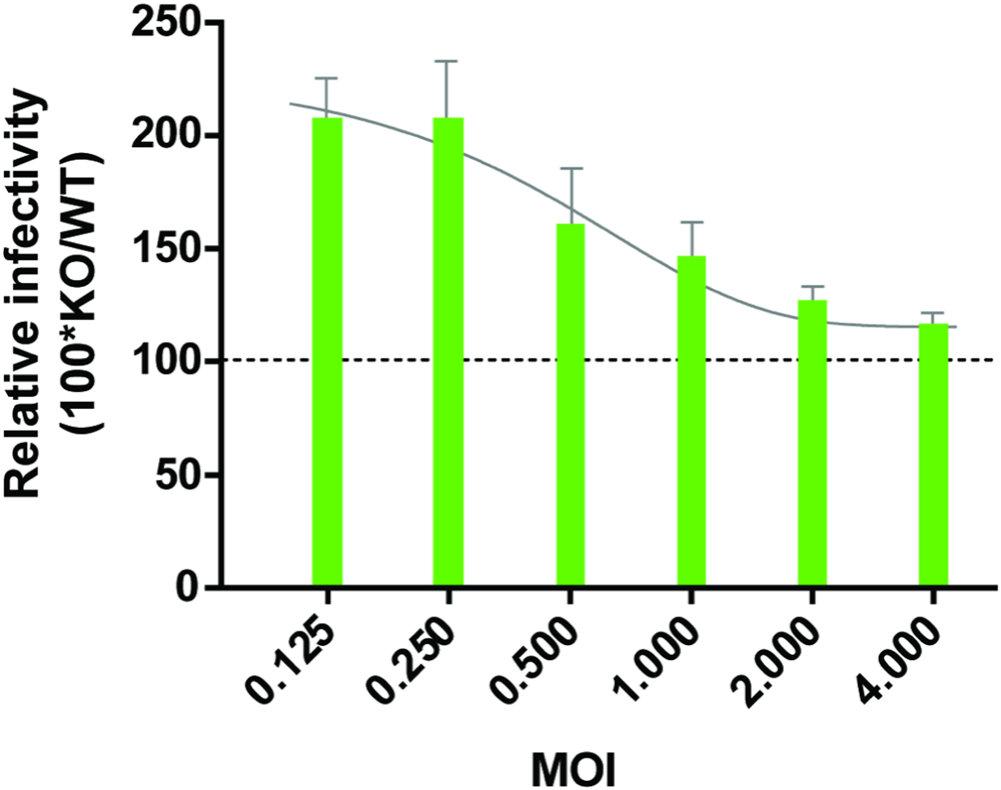
Infectivity of a MLV-based vector in GCN2^−/−^ and CGN2^+/+^ MEF cells. MEF cells (WT or GCN2^−/−^) were transduced with M.O.I. from 0.125 to 4. The percentage of transduced cells was determined by flow cytometry. For each point, the relative infectivity was determined as the level of GFP+ cells in GCN2^−/−^ MEFs normalized to the level in GCN2^+/+^ MEFs.

Finally, we addressed the question whether the control of infectivity was restricted to HIV-1 or could be extended to MLV. GCN2^+/+^ and GCN2^−/−^ cells were transduced with different concentrations of MLV particles. As observed in the case of HIV-1, the percentage of EGFP positive cells was significantly higher in GCN2^−/−^ cells than in GCN2^+/+^ cells at any given MOI (Fig. 10). This indicates that GCN2 is able to phosphorylate IN from HIV-1 and MLV, which in turn restricts lentiviral as well as gammaretroviral infections.

## Discussion

One of the new challenges in HIV research is to better understand the integration process at the cellular level including the mechanisms governing its regulation. While several post-translational modifications of HIV-1 IN have already been described^5^, there is to date no biochemical data on how IN is recognized and modified by these cellular factors. In the present study, we investigated how IN interaction with GCN2 led to the phosphorylation of the viral enzyme. Indeed, we found that GCN2 not only bound HIV-1 IN but recognized it as a substrate and catalyzed its phosphorylation. GCN2 has been extensively studied in yeast and human. It recognizes its substrate, eIF2α, through a _79_KGYID_83_ motif that is critical for the phosphorylation of a serine residue located at approximately 15 Å^35^. An extensive mutagenesis analysis also showed that the ultimate aspartic acid residue D_83_ of this motif is crucial. Only the mutant eIF2α harboring a conservative variation to a glutamic acid (KGYIE) retained the capability to be phosphorylated by GCN2. Comparing the primary sequence of HIV-1 IN and eIF2α showed that the eIF2α phosphorylatable residue S_51_ could correspond to HIV-1 IN residue S_57_, located at approximately 17 Å from _82_GYIE_85_ in the model of the HIV-1 IN structure^36^. Then, S_57_ showed to be phosphorylated by JNK^6^ may have been a good candidate to serve as the phosphorylation site for GCN2. However, the work by Manganaro *et al*.^6^ showed that phosphorylation of IN on residue S_57_ increased the protein stability leading to an increase in infectivity in activated T cells. In our study, infectivity was increased when phosphorylation of IN was prevented, either by mutating IN or by knocking out GCN2. These opposite phenotypes tend to argue that two different regulation mechanisms are involved in the work by Manganaro and our work.

Using various truncated versions of HIV-1 IN showed that only the CCD-CTD two domain fragment was an appropriate substrate for GCN2. Because neither the isolated CCD nor the CTD were efficiently phosphorylated by GCN2, this indicates that i) the interaction IN-GCN2 and the phosphorylation site are located at the CCD-CTD junction or distributed within the two domains, or ii) GCN2 recognizes IN under a specific oligomeric organization that can only be formed in the context of this two-domain fragment and not with the isolated domains. Using point mutations that affected the oligomerization status of IN also affected the phosphorylation of the mutant. This data is in favor of the second hypothesis where GCN2 would recognize a specific oligomer of IN. Similarly, using the PFV CCD with the HIV CTD enabled a phosphorylation of the chimeric protein at a level comparable to that of the full-length HIV-1 IN showing that the CCD of PFV can be used as a substitute to that of HIV-1. Thus, these data indicate that an IN CCD, either from PFV or HIV, was required to catalyze the GCN2 mediated phosphorylation. While we cannot rule that sequences conserved between the two orthologous proteins might be involved, it seems that the CCD might play a structural role required for GCN2 recognition. On the other side, the CCD of PFV IN only enabled phosphorylation by GCN2 of the CTD of HIV-1 IN and not of the native PFV IN CTD. Thus, it seems that the CTD of PFV IN is lacking an important feature for GCN2 activity. When comparing IN from HIV-1 and PFV, only 2 phosphorylatable residues serine or threonine are present in the CTD of HIV-1 IN and absent in that of PFV IN. Namely, HIV-1 IN harbors a serine in positions 255 and 283 while PFV IN harbors a glycine and histidine at the corresponding position, respectively. On the other hand, IN from HIV-2, ASV and MLV also sustained phosphorylation by GCN2. All these three proteins present a threonine residue at position 255 or position 359 in the case of MLV. Mass spectrometry confirmed that HIV-1 IN is indeed phosphorylated on two highly conserved serine residues, S24 and S255. Additional biochemical data obtained with mutants INs pinpointed to S255 as being the main phosphorylation site. Thus, it seems that GCN2 recognizes a specific 3D arrangement of the IN oligomeric complex as well as a specific sequence/residue located in the CTD of retroviral integrases.

In the present study, the infectivity of HIV-1 was increased in cells knocked out for GCN2. This suggested that when it is present, GCN2 can restrict viral replication. A similar phenotype was observed in our previous study, where cellular expression of GCN2 was decreased by RNA silencing^8^. But, having identified IN residues that were phosphorylated by GCN2, the corresponding mutations were introduced in the viral context and resulted in an increase in viral infectivity. Conversely, infectivity of viruses carrying an aspartic acid at IN residue 255, that mimics a phosphorylation, was decreased in GCN2^−/−^ cells compared to the WT. Although reverse transcription was not affected, viruses carrying mutations of GCN2’s phosphorylation site exhibited an increase in integrated DNA copy number. Together, these results suggest that GCN2 might decrease viral infectivity by limiting viral integration through the phosphorylation of IN residue S255.

Phosphorylation and proteolysis have already been linked in the case of enzymes that as IN belong to the family of the polynucleotidyl transferases, namely transposases. Additionally, it has been shown that IN from an avian retrovirus was phosphorylated on its CTD, on residue S282 and that phosphorylation could control the IN proteolytic processing^37, 38^. Here, no difference could be observed in term of stability, cellular expression or subcellular localization between the WT and mutant HIV-1 IN proteins over-expressed in 293 T cells. Thus, IN phosphorylation seems to restrict viral integration and replication without affecting IN bioavailability.

Still, the observed decrease in integration when IN is phosphorylated may arise from an interference with the interaction between IN and a cellular co-factor. This hypothesis is particularly attractive as a precedent has already been reported in the literature. In the case of the yeast Ty5 retrotransposon, Dai *et al*. showed that the Ty5 IN phosphorylation is required for the interaction of IN with the heterochromatin cellular protein Sir4, which tethers the integration into telomeres^39^. In the case of HIV-1, it is known that integration occurs mainly in the body of highly transcribed genes and that this selectivity is mainly driven by the interaction of IN with the cellular cofactor LEDGF. Sequencing of integration sites revealed that phosphorylation of HIV-1 IN doesn’t affect tethering of the pre-integration complex to the integration site. IN mutations did not affect the integration profile, in terms of genomic region (body of genes) or specific histone modifications. Yet, it should be pointed out that the comparison was performed using data from K562 and Gm12878 cells for the epigenetic marks map while our integration map was obtained in 293 T cells. Also, the S255D single mutant could not be analyzed because for an unknown reason we could not generate enough data.

Finally, the observed increase in viral infectivity when blocking IN phosphorylation might be the resulting of a direct effect of IN capability to catalyze integration. *In vitro*, various assays were performed to evaluate the potential effect of IN mutations on the 3′-P activity and the ST activity. While no significant differences were observed between non-phosphorylatable proteins and the WT enzyme, mutation S255D in combination or not with S24D induced a more profound catalytic defect. This is in agreement with the decreased level of integrated DNA observed with the S255D mutant virus and the corresponding increase obtained in the cells lacking GCN2. Altogether, the data showed that phosphorylating IN at position S255 might decrease its activity leading in the cell to lower level of proviral integration.

As first suggested by the *in vitro* phosphorylation of IN from other *orthoretrovirinae* such as MLV and ASV, GCN2 seemed to also restrict MLV infection. Similarly to what was observed in the case of HIV-1, infectivity of MLV-based viruses was enhanced in GCN2^−/−^ cells compared to a parental cell line, with a 2-fold increase in EGFP positive cells. Accordingly, it seems possible for GCN2 to control the replication of retroviruses for which the IN is phosphorylated by the kinase. Because retroviral vectors are actively studied for gene therapy, the fact that their efficiency may be directly linked to the capacity of GCN2 to phosphorylate the IN protein used in such a vector is of interest.

In conclusion, phosphorylation of enzymes involved in DNA mobility such as retroviral INs, eukaryotic transposases^40^ and yeast INs^39^, has been reported. Further studies will establish if GCN2 may exert a general control regulating DNA mobility in the cell and might consequently contribute to the general genome stability and innate immune response.

## Electronic supplementary material

Supplemental Table S1
