# Supplementary material for: GCN2 phosphorylates HIV-1 integrase and decreases HIV-1 replication by limiting viral integration
Source: Sci Rep. 2017 May 23;7:2283. doi: 10.1038/s41598-017-02276-0 (PMC5442153; doi:10.1038/s41598-017-02276-0)
Supplement: Supplementary file 1 — Supplemental Table S1 [file 41598_2017_2276_MOESM1_ESM.doc]

# GCN2 phosphorylates HIV-1 integrase and decreases HIV-1 replication by limiting viral integration

A Jaspart, C Calmels, O Cosnefroy, P Bellecave, P Pinson, S Claverol, V Guyonnet-Dupérat, B Dartigues, MS Benleulmi, E Mauro, PA Gretteau, V Parissi, M Métifiot* and ML Andreola^*^

| **Origin** | **Context** | **Property** | **Confidence** | **Sequence** | **Activation Type** | **Modifications** | **Residue** | **XCorr** | **ΔScore** | **Rank** | **Search Engine** | **Charge** | **Precursor m/ z [Da]** | **ΔM [ppm]** | **First Scan** | **Last Scan** |
| --- | --- | --- | --- | --- | --- | --- | --- | --- | --- | --- | --- | --- | --- | --- | --- | --- |
| BL21 | *in vitro assay with purified GCN2* | 1-288 | High | GEGAVVIQDNsDIKVVPR | CID | S11(Phosphorylation) | IN S255 | 3.09 | 1 | 1 | Sequest HT (13) | 2 | 988.50024 | 4.18 | 4388 | 4388 |
|  |  |  | High | GEGAVVIQDNsDIKVVPR | CID | S11(Phosphorylation) | IN S255 | 2.44 | 1 | 1 | Sequest HT (13) | 3 | 659.33551 | 3.56 | 4427 | 4427 |
|  |  |  | High | AmVsDFNLPPVVAK | CID | M2(Oxidation) S4(Phosphorylation) | IN M22/S24 | 2.28 | 1 | 1 | Sequest HT (13) | 2 | 792.38373 | 3.9 | 5984 | 5984 |
|  |  |  | High | AMVsDFNLPPVVAK | CID | S4(Phosphorylation) | IN S24 | 2.22 | 1 | 1 | Sequest HT (13) | 2 | 784.38574 | 3.27 | 6406 | 6406 |
|  |  | 50-288 | High | GEGAVVIQDNsDIK | CID | S11(Phosphorylation) | IN S255 | 2.75 | 1 | 1 | Sequest HT (13) | 2 | 762.85413 | 4.43 | 3475 | 3475 |
|  |  | 1-212 | High | AmAsDFNLPPVVAK | CID | M2(Oxidation) S4(Phosphorylation) | IN M22/S24 | 3.17 | 1 | 1 | Sequest HT (13) | 2 | 778.36804 | 3.93 | 4629 | 4629 |
| 293T | human cells expression | fused to EGFP | High | GEGAVVIQDNsDIK | CID | S11(Phosphorylation) | IN S255 | 2.75 | 1 | 1 | Sequest HT (2) | 2 | 762.85162 | 1.15 | 3307 | 3307 |
|  |  |  | High | FEGDtLVNR | CID | T5(Phosphorylation) | EGFP T119 | 2.24 | 1 | 1 | Sequest HT (2) | 2 | 565.74756 | 0.01 | 3476 | 3476 |
|  |  |  | High | AMAsDFNLPPVVAK | CID | S4(Phosphorylation) | IN S24 | 2.1 | 1 | 1 | Sequest HT (2) | 2 | 770.36804 | 0.67 | 6456 | 6456 |

**Supplementary Table S1. Identification by LC MS-MS of phosphorylated peptides of IN.** *In vitro* phosphorylation assay using HIV-1 IN full-length (1-288) or truncated variants were performed using recombinant GCN2 (BL21)*.* Alternatively, full-length IN fused to EGFP was expressed in human cells (293T) and pull-down using an anti-EGFP antibody. Proteins were analyzed by SDS-PAGE followed by silver nitrate staining, and bands corresponding to IN were analyzed by LC-MSMS. Hit corresponding to phosphorylated peptides are presented. The peptide FEGDtLVNR corresponds to a phosphorylated fragment corresponding to the EGFP region of the fusion protein.
